# Mélanome sur naevus congénital: à propos d'un cas

**DOI:** 10.11604/pamj.2014.19.161.4941

**Published:** 2014-10-16

**Authors:** Iman Hadj, Fatima Zahra Mernissi

**Affiliations:** 1 Service de Dermatologie, CHU Hassan II, Fès, Maroc

**Keywords:** Naevus congénital, mélanome, antécédents pathologiques, Congenital nevi, melanome, pathological history

## Image en medicine

Les naevus congénitaux surviennent chez environ 1% des nouveaunés. La classification la plus utilisée est basée sur leur taille; on parle de naevus congénital de petite taille quand le plus grand diamètre est inférieur à 1,5cm, de naevus de taille intermédiaire pour ceux compris entre 1,5 et 19,9cm, de naevus large quand le diamètre est supérieur à 20cm et de naevus géant quand la taille est supérieur à 40cm, avec fréquence des lésions satellites. Le risque de transformation en mélanome est estimé 6,3% pour les naevus géants, elle survient généralement avant l’âge de 20 ans, d'où l'indication d'une exérèse précoce. Ce risque est d'autant plus faible que la taille du naevus est petite, et la transformation est généralement tardive; Nous rapportons le cas d'une patiente de 60 ans, sans antécédents pathologiques, qui présente depuis la naissance un naevus congénital de la fesse gauche avec apparition il ya 2 ans d'un nodule pigmenté au sein de l'ancienne lésion augmentant progressivement de taille, l'examen dermato trouve un naevus congénital fusiforme de 12 cm de grand axe surmontée d'un tumeur pigmentée sessile mesurant 4 cm de diamètre, l'examen dermoscopique objective une vascularisation polymorphe avec un voile gris bleu, le reste de l'examen somatique trouve une adénopathie inguinale gauche centimétrique, l’étude histologique est en faveur d'un mélanome, le bilan d'extension objectivait une adénopathie inguinale suspecte dont l’étude histologique était en faveur d'une métastase. La patiente a bénéficié d'une exérèse avec des marges de 2 cm, puis adressée à l'oncologie pour prise en charge.

**Figure 1:**
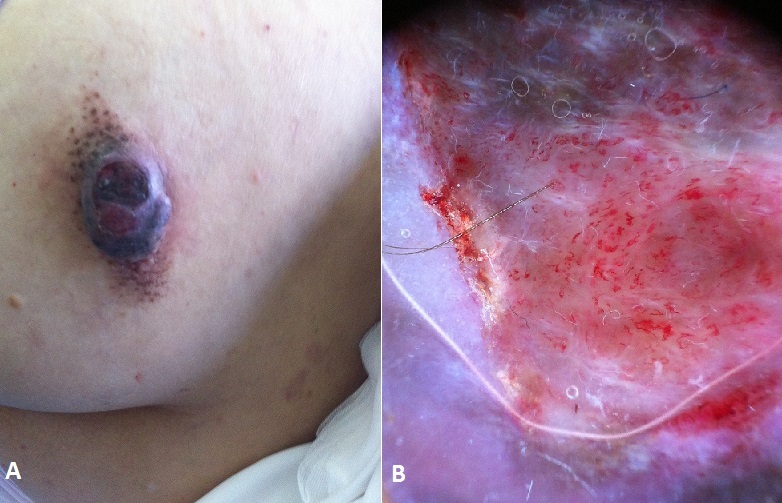
(A) naevus congénital fusiforme de 12 cm de grand axe surmontée d'une tumeur pigmentée sessile mesurant 4 cm de diamètre. (B) vascularisation polymorphe avec un voile gris bleu

